# IMPARO: inferring microbial interactions through parameter optimisation

**DOI:** 10.1186/s12860-020-00269-y

**Published:** 2020-08-19

**Authors:** Rajith Vidanaarachchi, Marnie Shaw, Sen-Lin Tang, Saman Halgamuge

**Affiliations:** 1 Research School of Electrical, Energy and Materials Engineering, College of Engineering & Computer Science, Australian National University, Acton, 2601 Australia; 2 Biodiversity Research Center, Academia Sinica, Nan-Kang, Taipei, 11529 Taiwan; 3 Department of Mechanical Engineering, University of Melbourne, Parkville, 3010 Australia

**Keywords:** Metagenomics, Inferring interactions, Network dynamics, Microbial interaction network

## Abstract

**Background:**

Microbial Interaction Networks (MINs) provide important information for understanding bacterial communities. MINs can be inferred by examining microbial abundance profiles. Abundance profiles are often interpreted with the Lotka Volterra model in research. However existing research fails to consider a biologically meaningful underlying mathematical model for MINs or to address the possibility of multiple solutions.

**Results:**

In this paper we present IMPARO, a method for inferring microbial interactions through parameter optimisation. We use biologically meaningful models for both the abundance profile, as well as the MIN. We show how multiple MINs could be inferred with similar reconstructed abundance profile accuracy, and argue that a unique solution is not always satisfactory. Using our method, we successfully inferred clear interactions in the gut microbiome which have been previously observed in in-vitro experiments.

**Conclusions:**

IMPARO was used to successfully infer microbial interactions in human microbiome samples as well as in a varied set of simulated data. The work also highlights the importance of considering multiple solutions for MINs.

## Background

Microbes are the most abundant, widespread organisms on Earth. They can be found in the biosphere, including all animals and plants, and most habitats in the oceans [1, 2], on land, or in air. Many studies show that microbes play a important role in the health and well-being of the hosts they are associated with. For example, in the human body, imbalances or changes in microbial communities correlates to various illnesses and other complications [3–9]. In plants, microbes provide essential nutrients, including all economic crops [10–12].

In the past, studying microbial communities through cultivation in laboratories was challenging [13]. Also, as over 99% [14, 15] of microbial species on earth are yet to be identified, the inability to cultivate and separate some microbial species in a laboratory environment have hindered progress on the study of microbiota.

Due to recent advances in 16S rRNA sequencing and high throughput sequencing, though, scientists can now explore the nature of real-world microbial samples and recognise individual species in these samples. 16S ribosomal RNA has been used by many scientists in order to identify, categorise and classify microbes.

Microbial networks are inherently complex in nature. With longitudinal studies, for example, it has become clear that the composition of microbial communities are constantly changing. Now, in order to properly understand these communities, it is important to study how they are changing, why they are changing, and how they interact with each other. To do so, it is important to acknowledge the following dynamics which play a part in the microbial composition changes. There could be temporal changes which are caused by external factors such as temperature variations [16], diurnal cycles [17] or seasonal variations [18]. In addition to these, other non-random co-occurrence patterns have been observed. Like in any other community, organisms in microbial communities interact in various ways with each other. Some of these interactions could be categorised under mutualism, competition, parasitism, predation, commensalism and amensalism [19].

Some important questions to ask about any biological community include, ‘Who is there?’, ‘What are they doing?’, and ‘How will they respond?’ [20]. While 16S ribosomal RNA sequencing can answer the first question, the latter two questions require an understanding of the interactions between different bacteria, hence the importance of inferring microbial interactions. These answers will improve our understanding of the human gut, the world’s oceans, plant root systems, lakes etc.

### Related work

With the advance of high throughput sequencing, high throughput inferring approaches have also been recently proposed. These are shown to be more successful than in-vitro analysis of interaction patterns [21]. Some of these approaches are Metagenomic Microbial Interaction Simulator (MetaMIS) [22], Rule-based Microbial Network (RMN) algorithm [23], Sparse Inverse Covariance Estimation for Ecological Association Inference (SPIEC-EASI) [24], Learning Interactions from Microbial Time Series (LIMITS) [25], Boolean Abundance Analysis [26], Boolean Dynamic Model [27], Stochastic Generalised Lotka-Volterra and Extended Kalman Filter (SgLV-EKF) [28] and Sparse Correlations for Compositional Data (SparCC) [29]. These algorithms mainly take two approaches [22], correlation-based analysis and model centred analysis. Often algorithms combine the two approaches to come up with a more robust method of inferring microbial interactions.

MetaMIS [22] uses a model-based approach where microbial interactions are assumed to abide by the biologically-inspired Lotka Volterra Model. The parameters of the Lotka Volterra model, which elucidate the interaction coefficients, are then approximated through a Partial Least Square Regression (PLSR). With these coefficients in place, the initial population is repopulated to recreate the community abundance profile. The accuracy metric is the Bray Curtis Dissimilarity between the original and recreated abundance profiles. The authors do not use any simulated data in their results but report inferences from male and female gut microbial communities. Their reported accuracy is 78% to 82%.

RMN [23] introduces its own model of Non-linear Regulatory OTU-triplet (NRO) model. This is a model for three OTUs which supposedly interact with each other. This assumption of interaction is then tested on the temporal abundance profile by a hyperbolic tangent based lack-of-fit function which they have introduced. The accuracy of the model is calculated based on correct inferences and correct non-inferences as a fraction of all inferences and non-inferences. Their reported accuracy is approximately 75% on simulated data. The authors use their method on infant gut data and infer previously known interactions.

SPIEC-EASI [24] is a correlation-based statistical method, which uses a Stability Approach to Regularisation Selection (STARS) to recreate the interaction correlations in form of a weighted undirected graph. Although this method does not indicate the nature of the interaction between two OTUs, it does give an idea of how close the OTUs are. The verification has been done through simulated data, and accuracy is measured with the Precision-Recall (P-R) curves and Area Under P-R Curves (AUPR). The authors have also presented the results from applying their method to the American Gut Project [30] data.

LIMITS [25], yet another model based algorithm, uses the discrete-time Lotka Volterra equations as the central microbial interaction model in its approach. The parameters of the Lotka-Volterra model is approximated through linear regression with an iterative bootstrapping approach. The verification is done through simulated data where the authors report a specificity of 60%–80% and a sensitivity of 70%–80%. They also analyse two individuals’ gut samples with the LIMITS algorithm. The major use of the LIMITS algorithm is to deduce keystone species.

Gao et al. [31], in their work, use a model based approach. They use a Lotka-Volterra model, fitted with abundance data using non-linear least squares minimisation technique. Then they use a forward step-wise regression method with bootstrap aggregation to select candidate models. These models are then filtered through a Bayesian information criterion which results in multiple models being selected. They aggregate the models into a single network as the output. The algorithm is tested on a cheese microbial community. The authors also apply the method on the gut microbiome of children with Type 1 diabetes. They do not present accuracy numerically, but confirm that their method was successful in inferring experimentally confirmed microbial interactions.

Boolean Analysis [26] uses an interesting model-based approach. The underlying biology is assumed to be forming either competitive links or synergistic links. Pairs of abundance vectors are analysed with the ESABO (Entropy Shifts on abundance vectors under Boolean operators) to confirm either a competitive or a synergystic link. Using a Jaccard index of the difference between the normalised number of correctly and incorrectly classified links, with their simulated data, they have achieved indexes ranging from 0.1–0.6 on competitive links and 0.1–0.9 on synergistic links. Their approach is also applied to a Human gut data-set.

Boolean Dynamic Model [27] does not contain an embedded biological model but assumes a binary relationship among OTUs. First, this method binarises the abundance data with a k-means binarisation, which allows binarisation with a threshold value, but with a stochastic element. Then it uses a recapitulating approach of updating and maintaining binary rules. The last part is a perturbation analysis, where it analyses the effects of removal (knock-out) or addition (forced overabundance) on the created model. This method is effective as for the work’s purpose of analysing *Clostridium difficile* infection in the gut. The finding is that *Barnesiella intestinihominis* hinders the growth of *Clostridium difficile*. This has been confirmed in in-vitro experiments.

SgLV-EKF [28] model is a straightforward approach of using the Lotka Volterra equations as the underlying biological model. But it improves the generalised Lotka Volterra system by introducing a Gaussian noise term, making it stochastic. Then the LV parameters are estimated using an Extended Kalman Filter (EKF), giving it the name SgLV-EKF. This algorithm is tested on Monte-Carlo simulated data, and shows an accuracy of 75%, with Mean Square Error (MSE) being the indicator of accuracy. The authors also apply the method on two mouse gut systems infected by *Clostridium difficile*, one being treated with clindamycin.

SparCC [29] is a co-occurrence based method which iteratively finds non-random co-occurrence patterns in microbial data. One of the first methods proposed in inferring microbial interactions, SparCC has shown a considerable improvement from Pearson Correlation method. On simulated data it has shown to achieve root mean squared errors (RMSE) as low as 0.02. The authors also apply the method on Human Microbiome Project data to show its usability on real life data.

Considering the literature, there seems to be a shift towards using model-based systems, with the support of statistical methods, rather than depending purely on statistical methods. An explanation of this is that, due to the complex nature of the microbial communities, purely mathematical methods, which ignore the underlying biology, would be prone to overlooking important biological constraints. Microbial communities have biologically specific behavioural dynamics, which cause non-independence between adjacent time-steps. Hence models which take into account these behavioural dynamics are useful in inferring the interactions.

On examining existing model based work, it is notable that Lotka Volterra Equations or one of its adaptations has been used in many approaches as the underlying biological model. The major reason for this use is that it has been shown that Lotka-Volterra Model can successfully simulate a microbial community when applied to different scenarios such as Lake Ecosystems [32], Human and murine intestinal microbial systems [33, 34] or the microbial ecosystem which occurs in the process of ripening of smear cheese [35]. The generalised Lotka Volterra equations have the capacity to capture the growth rates and the pairwise interactions of the OTUs, which are the important coefficients estimated in the process of inferring Microbial Interaction Networks (MINs).

Many of these studies have applied new methodology to simulated data as well as real-life data. This is important because data simulations always assume a known biological model, and the inherent noise in a biological system is not always present in artificially simulated data. Our work and the majority of other works are also guilty of using the same biological model in the inference algorithms, as well as in the data simulations. Hence some sort of verification with real-life data is obviously important. The problem with using real-life data for verification is that sans in-vitro studies, it is difficult to discern whether the inferred interactions are in fact bona fide interactions found in that microbial system. One potentially useful verification strategy is to highlight the overlap between identified interactions and interactions that were previously known. MetaMIS [22] uses an abundance profile reconstruction strategy to confirm their results. This system of verification influenced our method.

### Motivation & contributions

**Fig. 1:**
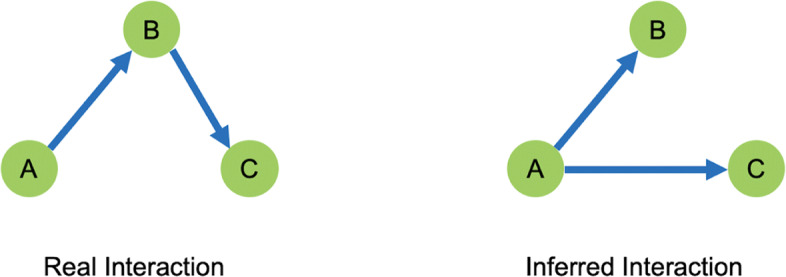
Although the real interactions are *A*→*B* and *B*→*C*, through A’s influence on B, A has an indirect influence on C. When these interactions are inferred through an abundance profile, the indirect interaction *A*→*C* may be inferred instead

It was interesting to note that the above mentioned methods imply a unique solution to the problem of inferring a microbial interaction network, given a particular abundance profile. In their work addressing pitfalls in inferring microbial dynamics, however, Cao et al. [36] demonstrate that multiple interaction networks can lead to the same abundance profile. This is supported by the simple scenario of three OTUs with indirect interactions, as shown in Fig. 1.

In this paper we present IMPARO (Inferring Microbial interactions through PARameter Optimisation), an algorithm for microbial interaction inference which incorporates biologically meaningful models for the interaction network as well as the abundance profile.

IMPARO is the first inference method to not make the assumption of a unique inferred solution, and to explore multiple solutions with similar accuracy levels. Because of the inherent noise in microbial abundance data, it is reasonably assumed that small changes in accuracy do not necessarily mean superior MINs.

It is also the first to assume an underlying biological model for a microbial interaction network (MIN), by using the microbial community dynamics model introduced in [37]. The shift from statistical methods to model-based methods was inspired by using an underlying biological model for the Abundance Profile, and models such as gLV, SgLV, NRO and entropy shift of competitive synergistic links were used. Our work goes a step further in introducing an underlying biological model for the MIN, which reduces the optimiser search space by pruning solutions which are less feasible biologically.

It also contains a Monte Carlo approach [38] for the purpose of encompassing the effect of rarer OTUs into the inferred MIN. Most statistical methods fail to do justice to the effects of rarer OTUs simply because their presence is overwhelmingly shadowed by the other OTUs. And most model based solutions use filtering processes which favour higher ranked (in terms of abundance) OTUs before the inference process. But in fact, the majority of OTUs in a community are rarer OTUs [22, 39].

Our results are verified through both simulated and real-life data. Our simulations take into account the diversity of microbial communities. Community dynamics models are used to ensure different types of communities are included in our testing. We compare the results from IMPARO with results reported in literature.

Key Contributions Summarised:

- Inference of interactions without the assumption of a unique solution.
- Consideration of an underlying biological model for the MIN.
- Using a Monte Carlo approach to ensure a better representation of rarer OTUs.
- Verification of the algorithm on real life and simulated data.
- Comparison of results with that of existing methods.

## Results

IMPARO was used to infer interaction parameters in both simulated and real life data. We present the overall results in this section. Additional results and snapshots of simulated data are available in Additional file 3.

### Simulated data

Data simulation was performed using the microbial community dynamics model described above, and focuses on heterogeneity and sparsity variation. Nominal component **N** is sampled from a normal distribution $\mathcal {N}(0, 1)$. Initial abundance values were sampled randomly from a uniform distribution $\mathcal {U}(0, 1)$, as suggested in [37]. In this study we are interested in examining how IMPARO handles data-sets with varying heterogeneity and sparsity. For the purpose of the simulated study, we used ten species.

For the heterogeneity study we use *P*(*α*) s.t. *α*∈[0.2,0.4,0.6,0.8,1.0], so that communities with a heterogeneity favouring a minority of highly influential OTUs are considered.

For the sparsity study we use *G*(*n*,*p*) s.t. *p*∈[0.2,0.4,0.6,0.8,1.0]. This would include communities which are very sparse (0.2) to fully connected (1.0).

The Mean Squared Error (MSE) between the ground truth and the inferred parameters in each case as described above are shown in Table 1. We observe that lower *p* values and higher *α* values—highly sparse and highly heterogeneous instances—result in lower errors.

**Table 1:** MSE values from the heterogeneity and sparsity study

*σ*=1		P				
		*p*=0.2	*p*=0.4	*p*=0.6	*p*=0.8	*p*=1.0
H	*α*=0.2	0.05	1.32	1.36	2.55	1.99
	*α*=0.4	0.61	0.63	1.36	0.66	1.02
	*α*=0.6	0.42	0.57	1.54	1.98	1.81
	*α*=0.8	0.09	0.57	1.14	0.79	1.51
	*α*=1.0	0.34	0.28	0.71	0.73	1.28

Heterogeneity and sparsity were varied—through varying *α* and *p* respectively—to investigate how IMPARO responded to microbial samples of varying nature. Mean Squared Error(MSE) indicates how far the inference is from the ground truth

Tested for robustness with Gaussian noise (*μ*=0.0, *σ*=0.01), IMPARO returns solution clusters which are within mean squared errors of 0.4 - 0.5 of each other, suggesting the solutions are robust.

### Existence of multiple solutions

As we have mentioned in the literature review, it is possible to find multiple solutions for the problem of inferring microbial interactions when the accuracy is measured through reconstructed abundance profiles.

**Fig. 2:**
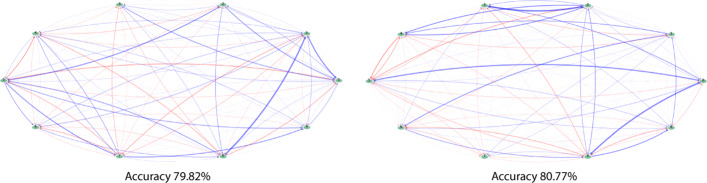
An example of two distinct solutions for the same simulated data-set. The MINs corresponding to each solution, when evaluated with reconstructed abundance profile accuracy were within 1% of each other

In Fig. 2 we present two MINs inferred from the same abundance profile, which—after recreating the abundance profile and measuring for accuracy using Bray-Curtis metric—returns accuracies within 0.1% (79.82% and 80.77% respectively). Compared to the true values used in simulating the data, they indicate mean squared errors of 0.59 and 0.58 respectively.

### Tests on real life data

For this study we use the data from human faecal microbiome samples collected from a healthy male and a female for time spans of 15 months and 6 months respectively [39]. This data is publicly available at MG-RAST:4457768.3-4459735.3.

On female faecal microbiome analysing the 10 highest ranking OTUs, our method achieves a 84.22% reconstructed abundance profile accuracy. On the male faecal microbiome OTU rankings, our method achieves a 81.60% accuracy. It should be noted that in the female sample, 185 time points were taken into account. In the male sample 442 time points were considered. In both instances the sparsity of the connections were assumed to be 50% for the inference process.

The results for the female faecal microbiome sample showing reconstructed abundance profile accuracy values for varying numbers of highest ranking OTUs are tabulated in Table 2.

**Table 2:** The results for the female faecal microbiome sample showing reconstructed abundance profile accuracy values for varying numbers of highest ranking OTUs

No of Highest Ranking OTUs	Reconstructed Abundance Profile Accuracy
5	85.42%
10	84.22%
20	82.77%
30	79.93%
40	81.86%
50	82.08%
60	74.83%
69	80.11%

As a further analysis, we inferred MINs at different taxonomic resolution levels—from Phylum to Genus. The reconstructed abundance profile values of this study performed on the female faecal microbiome is tabulated in Table 3. The ten highest ranking OTUs were considered in this study.

**Table 3:** Inspecting the reconstructed abundance profile accuracy with varying taxonomic resolution levels in the female faecal microbiome

Taxonomic Resolution Level	Reconstructed Abundance Profile Accuracy
Genus	76.30%
Family	84.22%
Order	87.22%
Class	87.54%
Phylum	87.63%

### Inference of rarer OTU interactions

In order to understand how our method works for rarer OTUs, we processed randomly selected samples from the female faecal microbiome with at least 50% of the considered OTUs from the rare range (average abundance lower than 0.1%). In some studies [22, 23] these rare OTUs are discarded while favouring the most abundant OTUs. But we show that rarer OTUs can indeed be considered in the inference process, and give satisfactory results. Our samples provided an average accuracy (reconstructed abundance profile accuracy) in the order of 60%.

## Discussion

In this section we analyse the results obtained by IMPARO.

### Simulated data

The simulated study indicates that, IMPARO works better with data samples with low heterogeneity and high sparsity (low *p* value). When considering highly heterogeneous samples, we attribute the larger errors to the difficulty in inferring near-zero values. For less sparse data-sets this can be attributed to the difficulty in inferring a fully connected MIN. The best case as seen in Table 1 being the most heterogeneous and sparsest instance can be attributed to it being close to the trivial case of all zeros. It is indeed expected to have better results in the more sparse samples, as GAs tend to converge faster when the dimensions of the parameter space are lower. Achieving better results on low heterogeneous and moderately sparse samples in the simulated data explain the better results obtained in real-life samples with the higher ranking OTUs, which are more homogeneous and are assumed to be moderately connected.

### Existence of multiple solutions

Although the reconstructed abundance profile accuracy is indicative of the prediction accuracy of the interaction parameters, there seems to be multiple distinct solutions for interaction matrices resulting in similar abundance profile accuracies. Also to be noted is that these distinct solutions are within 1–2% of reconstructed abundance profile accuracy. Because of the high noise in microbial data-sets, a solution which is only 1–2% better in recreated abundance profile accuracy cannot be considered to be a superior solution. A possible cause for multiple solutions could be the optimiser being stuck at local optima. However as the parameter space has too many dimensions to permit visualisation, the methods need to rely on results obtained from multiple initialisations. While recognising GA is particularly challenged with overcoming local optima, it is worth looking into other explanations possible. One cause for multiple distinct solutions is the possibility that indirect interactions are being inferred incorrectly through these methods.

We may conclude that good reconstructed abundance profile accuracy is a necessary condition for a precise prediction although it is not a sufficient condition by itself. Hence we highlight the need to widen the search for all such instances where the reconstructed abundance profile accuracy is higher than a threshold value. An optimisation approach which provides multiple answers is, therefore, important.

### Tests on real life data

First we note that the inference of the male faecal microbiome resulted in a lower accuracy compared to the female faecal microbiome. This might be due to the fact that male sample covers a greater time period than the female sample. (442 time points over 15 months in comparison to 185 time points over 6 months).

Apart from the increased difficulty in predicting a longer time series, it can also be hypothesised that the inherent changes in the microbiome itself over a longer period of time could be a reason for the reduced predictive accuracy. Microbes, as any other community of living organisms, change over time, which includes changes in the nature of their interactions.

In Table 2 we observe a trend towards the accuracy decreasing as the number of OTUs included is increased. The reasons for this could be two-fold. Firstly, as the number of OTUs increase, the number of parameters to be estimated grows quadratically. Secondly, as more lower ranked—and rarer—OTUs are considered, the difficulty level of inference increases.

We observe that higher accuracy levels correspond to higher taxonomic ranks in Table 3. Considering that the number of OTUs remained constant in this study, we conjecture that as abundances get more numerous for each OTU with each higher taxonomy level, abundance profiles become less disorderly. This could have resulted in better reconstructed abundance profile accuracies for higher taxonomic resolution levels.

**Fig. 3:**
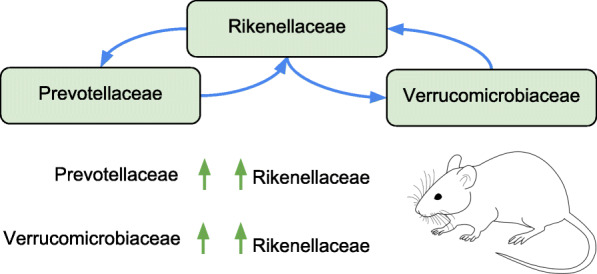
Strong microbial interactions inferred from the female faecal microbiome have been previously observed in in-vitro studies of murine microbiome

Of mutualism interactions inferred by our algorithm, some have been shown to exist in previous studies as shown in Fig. 3. The population of bacterial families of *Prevotellaceae* and *Rikenellaceae* has shown to increase simultaneously in immune impaired Nod2(-/-) mice faecal microbiome [40]. The populations of *Rikenellaceae* and *Verrucomicrobiaceae* have been shown to simultaneously increase in another study of mice faecal bacteria studying diet induced obesity [41]. Both these results were inferred from the female faecal microbiome sample.

### Consideration of rarer OTUs

From the results, it could be seen that when the rarer OTUs are taken into account, the predictive power is significantly less. Even though the predictive power is less, the approximately 60% reconstructed abundance profile accuracy suggests promise in exploring the question of inferring interactions for rarer OTUs further. Also, when combined with higher ranking OTUs, rarer OTUs do not significantly reduce the accuracy of the whole sample, as indicative from the results in Table 2.

### Analysis of errors

We consider the reasons for 20% error margin of IMPARO to be threefold. Firstly, microbial interactions are prone to change over time. When interactions are inferred over multiple points covering a large time interval, this could add a significant error. Secondly, the high dimensionality of the search space increases the chance of local optima, thus resulting in higher errors. Thirdly, as the input data is acquired through experimental means, we expect the errors from the experimental procedures and data collection to have contributed to the overall error.

### Future work

There are several possible ways of extending IMPARO, to alleviate some of its weaknesses. IMPARO attempts to infer a single interaction parameter for each OTU couple for the entire time-line. We note that, as microbial interactions are prone to change over time, it can be beneficial to infer interactions over separate time intervals, which could allow better abundance profile recreation and exploration of interaction parameter dynamics over time. Also IMPARO currently lags at inferring rarer OTUs, as compared to higher ranking OTUs. Supplementing genomic data with transcriptomic data in the inference process can potentially increase the prediction quality. It is also worth exploring how IMPARO can be improved to deter the disruption of the community dynamics model by zero and non-zero values.

## Conclusions

Inferring microbial interactions will advance our understanding of microbial communities. We have presented IMPARO, a microbial interaction inference algorithm based on parameter optimisation. We have conducted studies on simulated microbial communities and on real-life data. IMPARO has shown to successfully infer interaction parameters corresponding to microbial systems in the human body. We also emphasise the importance of considering multiple solutions for the MINs.

## Methods

In this section we present the methods used in IMPARO.

### Generalised Lotka Volterra model

The Generalised Lotka-Volterra Model (GLV) is a system of Ordinary Differential Equations. In inferring interactions the GLV is used in its discrete form, where each time point represents a sample in the temporal abundance profile. The differential equations describe the difference of a single OTUs abundance levels in two adjacent time points, and how it is dependant on the growth rate and its interaction coefficients with the other OTUs.

1 $$  \frac{d}{dt}x_{i} (t_{k}) = r_{i}x_{i}(t_{k}) + x_{i}(t_{k}) \sum^{L}_{j=1} A_{ij}x_{j}(t_{k})  $$

In Eq. 1*x*_*i*_(*t*_*k*_) describes the relative abundance of the *i*^*th*^ OTU at time *t*_*k*_. The growth rate of the *i*^*th*^ OTU is described by *r*_*i*_. **A** is the overall interspecific interaction matrix, where **A**_*ij*_ describes the effect on the *j*^*th*^ OTU by the *i*^*th*^ OTU. (**A**_*ij*_<0 represents a negative effect on the *j*^*th*^ OTU by the *i*^*th*^ OTU). The saturation terms have not been included as we do not consider communities to have known carrying capacities.

We use the above framework as it is in our implementation and add a noise term afterwards to compensate for inherent and experimental noise in microbial data. All the abundance values are normalised for each time point.

### Community dynamics model

Introduced by Gibson et al. [37], the community dynamics model is best described as a Mathematical Model consisting of set of Matrices which represent different qualities in microbial interactions.

2 $$  A = NH \circ Gs  $$

In Eq. 2**A** is the microbial interaction matrix, **N** is the nominal interspecific interaction matrix, **H** is the heterogeneity matrix and **G** is the adjacency matrix of the underlying ecological network. *s* is a scaling coefficient. The operator ∘ represents the Hadamard product (element-wise multiplication of matrices).

$\mathbf {N}\in \mathbb {R}^{n \times n}$, the nominal interspecific interaction matrix has a normal distribution with a mean of 0, and a standard deviation of *σ*^2^, i.e. $\mathbf {N}_{ij} \sim \mathcal {N}(0, \sigma ^{2})$. This matrix warrants that the interactions are fair in the absence of an influencing factor, which is introduced in the next component. $\mathbf {H} \in \mathbb {R}^{n \times n}$, the heterogeneity matrix is a diagonal matrix with a power-law distribution, with an exponent of *α*, i.e. $\mathbf {H}_{ii} \sim \mathcal {P}(\alpha)$. This matrix simulates the difference in the interspecific influence levels. It is believed that in a typical community there are a small number of highly influential species [42]. Together with the interspecific interaction matrix, the heterogeneity matrix assures a balanced community dynamics model. The next step is defining the connectedness, as MINs are generally not fully connected but sparse. $\mathbf {G} \in \mathbb {R}^{n \times n}$ is a binary matrix where **G**_*ij*_=1 represents that the OTU *i* is affected by OTU *j* and **G**_*ij*_=0 represents otherwise. This matrix follows an Erdős–Rényi model with *G*(*n*,*p*) where *n* is the number of OTUs and *p* is the probability of an edge which also represents the sparsity of **G**. (An illustrated numerical example is given in Additional file 1.)

### Bray Curtis dissimilarity

Bray-Curtis dissimilarity [43] is used in our work to determine the dissimilarity between two samples, specifically the dissimilarity between corresponding time-points in original and recreated abundance profiles. However a limitation of using the Bray Curtis Dissimilarity is that the dissimilarity metric is biased towards more abundant species.

3 $$  BCD(\mathbf{x}_{(t_{k})}, \mathbf{x}^{*}_{(t_{k})}) = \frac{\sum_{i=1}^{L}\left | x_{i_{(t_{k})}} - x^{*}_{i_{(t_{k})}} \right |}{ \sum_{i=1}^{L} \left(x_{i_{(t_{k})}} + x^{*}_{i_{(t_{k})}} \right)}  $$

4 $$  BCD_{overall} = \frac{\sum_{k=0}^{T} BCD(\mathbf{x}_{(t_{k})}, \mathbf{x}^{*}_{(t_{k})})}{T}  $$

where $\mathbf {x}_{(t_{k})}$ and $\mathbf {x}^{*}_{(t_{k})}$ represent relative abundances of the original and recreated abundance profile, at time *k*. $x_{i_{(t_{k})}}$ represents the relative abundance of the *i*^*th*^ OTU of the original abundance profile at time point *k* and $x^{*}_{i_{(t_{k})}}$ represents the same in the recreated abundance profile. *L* is the number of OTUs in the sample, while *T* is the total number of time-points in the abundance profile.

### Reconstructed abundance profile accuracy

The reconstructed abundance profile accuracy is a metric of how accurately the original abundance profile can be reconstructed with the inferred MIN. Using the original initial conditions, $\mathbf {x}_{(t_{0})}$, the subsequent microbial community compositions are calculated using the generalised Lotka-Volterra model. This reconstructed microbial community abundance profile is then compared to the original abundance profile using the Bray Curtis Dissimilarity. This metric reflects the quality of the inferred MIN.

### Kolmogorov-Smirnov test

We use the Kolmogorov-Smirnov Test as a goodness-of-fit test to compare the empirical distribution of the inferred MIN to a model empirical distribution which follows the Community Dynamics Model.

5 $$  D_{n,m} = \sup_{x}|F_{1, n}(x) - F_{2, m}(x)|  $$

where *F*_1,*n*_(*x*) and *F*_2,*m*_(*x*) are the empirical distribution functions for the parameters of the microbial interaction networks. Here parameters of the interaction networks are considered as one-dimensional probability distributions. (i.e. each interaction is considered to be independent). sup is the supremum function [44].

### Inferring MINs from abundance profile

We are viewing the inference of MINs as an optimisation problem. As our aim is to estimate the elements of the matrix **A**, the overall interspecific interaction matrix, this can specifically be described as a large parameter optimisation problem, because the parameters we are estimating is in the order of *N*^2^, where *N* is the number of OTUs taken into consideration. The interaction coefficients of the bacteria community are considered to be the parameters. In the simplest case, the value we are optimising is the averaged Bray-Curtis Dissimilarity over the time axis, for the original abundance profile and the recreated abundance profile from generated with the parameters. We later take the statistical similarity of the parameter set (interaction coefficients) to the theoretical distribution of interaction coefficients according to the microbial community model.

MINs are estimated to be sparse in nature [45]. This information can be used to our advantage in optimising the parameters because the adjacency matrix of a sparse MIN contains many zero values. But what we do not know is which parameters should be set to zero, and which parameters should be set a non-zero value. Here we use a Genetic Algorithm (GA) [46, 47] based approach whose Monte-Carlo simulation of Adjacency Matrices for MINs allow an estimated percentage of values to be set to zero, and to reevaluate that based on the BCD, which we are trying to minimise.

For the purpose of the GA, we consider each element in the matrix **A** to be a gene, and a collection of elements to be a chromosome. Because we are expecting sparse MINs, the chromosomes do not contain *N*^2^ number of genes. This reduces the computational complexity. The algorithm makes mutations to the genes, which affect both row (*i*), column (*j*), and numeric effect (**A**_*ij*_). The crossover operation is a single-point crossover, where a randomly selected part of a single chromosome is replaced by the corresponding part of another chromosome.

The algorithm uses a two-fold fitness function where a score is assigned to each chromosome based on the BCD and a penalty is assigned based on the likelihood of being compatible with the community dynamics model. Thus, our algorithm considers underlying biological compatibility for both the abundance profile - in terms of OTU propagation through the generalised Lotka Volterra Equations, and the Adjacency Matrix for MIN with the community dynamics model.

**Fig. 4:**
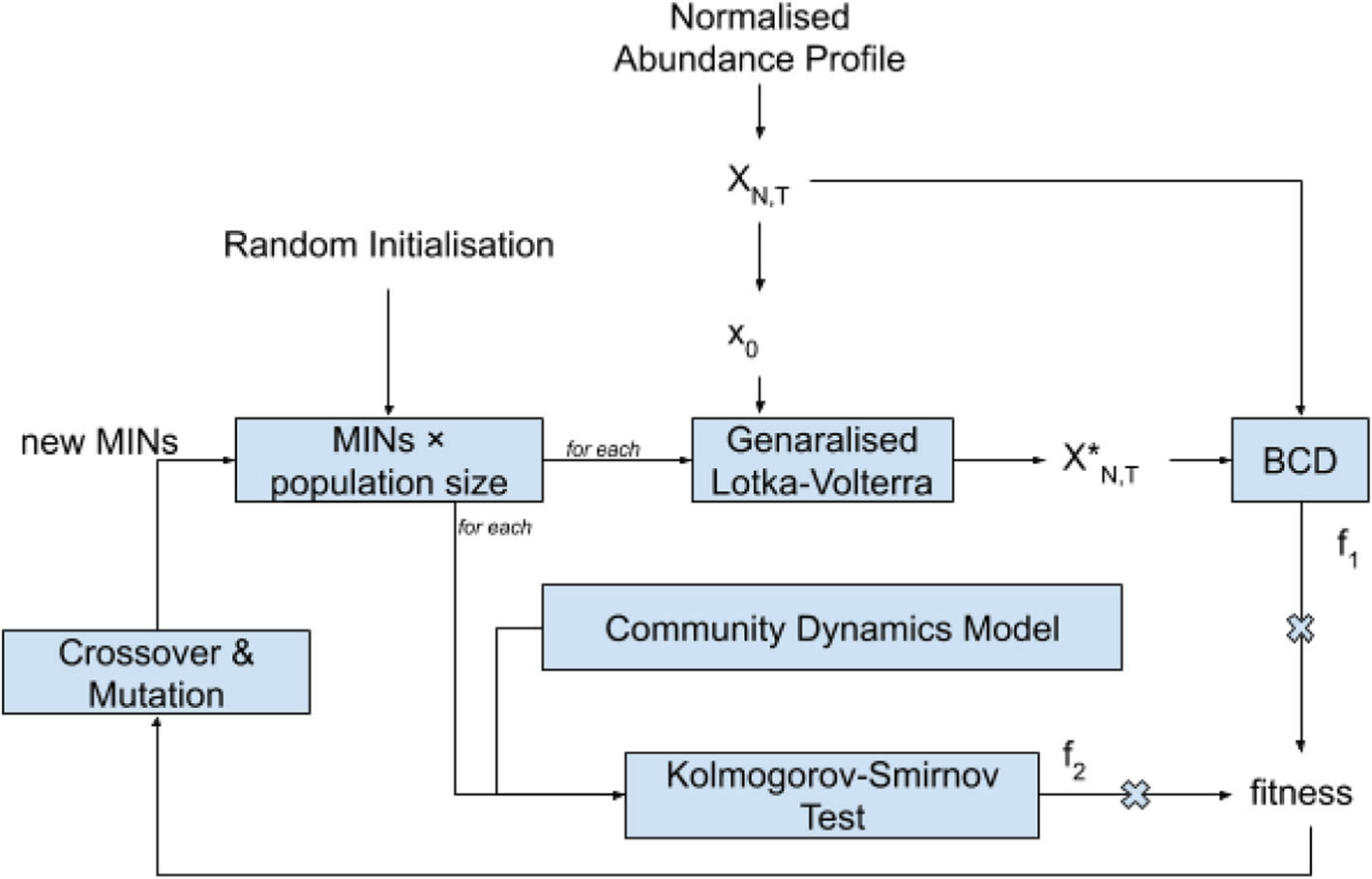
The process of IMPARO includes a Genetic Algorithm, which takes into account the Bray-Curtis Dissimilarity (BCD) and the Kolmogorov-Smirnov Test to calculate the fitness of a solution. The combined score ensures that the Microbial Interaction Networks (MINs) provided by the algorithm are feasible solutions. *X*_*N*,*T*_ is the microbial abundance profile, with *N* OTUs, and *T* time points. *X*_0_ is the microbial abundances at the initial time point. $X^{*}_{N,T}$, is the recreated abundance profile. *f*_1_ and *f*_2_ respectively are the factors BCD and K-S Test scores counting towards the overall score

The first part of the score is straightforward, with the BCD. For the penalisation step, it is important to explore the probability distributions of the community dynamics model. The matrix **A**’s near zero values are identified and zeroed at first, to satisfy sparseness. The generated matrix is checked for compliance with expected statistical properties using the Kolmogorov-Smirnov (KS) test, and penalties are applied according to the KS statistic [44]. Thus a combination score makes sure that future generations of solutions are compatible with the underlying biological models in terms of MIN and abundance profile. This process is illustrated in Fig. 4. Important code segments are provided in Additional file 2.

The GA approach in IMPARO which uses Monte Carlo methods for gene introduction allows rarer OTUs a better representation in the solution.

## Supplementary information

**Additional file 1** Illustrated Example of Community Dynamics Model

**Additional file 2** Details of the Genetic Algorithm

**Additional file 3** Results and Data Snapshots

## Data Availability

Real-life data used in this study is publicly available at MG-RAST:4457768.3-4459735.3. Snapshots of simulated data are provided in the Additional file 3. The code of IMPARO is available at https://bitbucket.org/rajith/imparo/, and is released publicly under MIT license. All simulated data sets are also available in the repository.

## Abbreviations

IMPARO: Inferring microbial interactions through parameter optimisation
LV: Lotka-Volterra
gLV: Generalised Lotka-Volterra
SgLV: Stochastic generalised Lotka-Volterra
NRO: Non-linear regulatory OTU-triplet
OTU: Operational taxonomic unit
MIN: Microbial interaction network
MSE: Mean squared error
