# Supplementary material for: IMPARO: inferring microbial interactions through parameter optimisation
Source: BMC Mol Cell Biol. 2020 Aug 19;21(Suppl 1):34. doi: 10.1186/s12860-020-00269-y (PMC7436957; doi:10.1186/s12860-020-00269-y)
Supplement: Supplementary file 1 — Additional file 1 Illustrated Example of Community Dynamics Model [file 12860_2020_269_MOESM1_ESM.pdf]

Supplementary Material for IMAPRO: Inferring Microbial Interactions through Parameter Optimization

Numeric Example of the Community Dynamics Model

$$A = NH \circ Gs$$

A - Overall Microbial Interaction Matrix
N - Nominal Interspecific Interaction Matrix
H - Heterogeneity Matrix
G - Adjacency Matrix of the underlying ecological network
s - scaling factor
◦ is the Hadamard Product

Numerical Example with four species:

$$N = \begin{bmatrix} 0 & 0.1 & 0.5 & -0.2 \\ 0.6 & 0 & 0.2 & 0.5 \\ -0.2 & 0.8 & 0 & 0.2 \\ -0.4 & -0.1 & 0.5 & 0 \end{bmatrix}$$

$$H = \begin{bmatrix} 9 & 0 & 0 & 0 \\ 0 & 0.3 & 0 & 0 \\ 0 & 0 & 0.4 & 0 \\ 0 & 0 & 0 & 0.5 \end{bmatrix}$$

$$G = \begin{bmatrix} 1 & 1 & 0 & 1 \\ 0 & 1 & 0 & 0 \\ 1 & 0 & 1 & 0 \\ 1 & 1 & 1 & 1 \end{bmatrix}$$

$$s = 1$$

Final Result:

$$A = \begin{bmatrix} 0 & 0.03 & 0.2 & -0.1 \\ 0 & 0 & 0 & 0.25 \\ -1.8 & 0 & 0 & 0 \\ -3.6 & -0.03 & 0.2 & 0 \end{bmatrix}$$

For more information please refer “Gibson et al., On the Origins and Control of Community Types in the Human Microbiome, PLOS Computational Biology”
