# Supplementary material for: IMPARO: inferring microbial interactions through parameter optimisation
Source: BMC Mol Cell Biol. 2020 Aug 19;21(Suppl 1):34. doi: 10.1186/s12860-020-00269-y (PMC7436957; doi:10.1186/s12860-020-00269-y)
Supplement: Supplementary file 2 — Additional file 2 Details of the Genetic Algorithm [file 12860_2020_269_MOESM2_ESM.pdf]

Supplementary Material for IMAPRO: Inferring Microbial Interactions through Parameter Optimization

Details of the Genetic Algorithm (GA)

Initialising the population

```
For each chromosome in population:
      For each gene in chromosome:
            gene.i <- RandomInt(0, n)
            gene.j <- RandomInt(0, n)
            gene.A <- RandomNormal( 0, std)

Each gene represented A[i, j], where A is the Microbial Interaction
Network
```

Mutation

```
For each gene in Chromosome:
      Where R is a Random(0, 1)
      If R < MutationChance:
            gene.i <- RandomInt(0, n)

      // Similar mutation for gene.j and gene.A
```

Crossover

```
Where CP is a RandomInt(0, length of the GenesList)

Chromosome1[0 to CP] <- Chromosome2[0 to CP]
```

Score Calculation

```
Let f1 be the factor from BCD
Let f2 be the factor from Community Dynamics Model

For the chromosome:

Calculate chromosome.A
Get Initial timepoints (X[0])  from the abundance profile

Run LV(chromosome.A, X[0]) to get the recreated abundance profile X
```

```
S1 = 1 - BCD(X, AP)

Create model community MIN from Community Dynamics Model
S2 = KS_Statistic(chromosome.A, Model.MIN)

Score = S1 x f1 + S2 x f2
```

Fitness

```
Crom_i.fitness = Chrom_i.Score /( sum of all chromosome scores)
```
