# Supplementary material for: IMPARO: inferring microbial interactions through parameter optimisation
Source: BMC Mol Cell Biol. 2020 Aug 19;21(Suppl 1):34. doi: 10.1186/s12860-020-00269-y (PMC7436957; doi:10.1186/s12860-020-00269-y)
Supplement: Supplementary file 3 — Additional file 3 Results and Data Snapshots [file 12860_2020_269_MOESM3_ESM.pdf]

Supplementary Material for IMAPRO: Inferring Microbial Interactions through Parameter Optimization

Additional Results & Data Snapshots

Female Faecal Microbiome Results

No of highest ranking OTUs included	Reconstructed Abundance Profile Accuracy
5	85.42%
10	84.22%
20	82.77%
30	79.93%
40	81.86%
50	82.08%
60	74.83%
69	80.11%

Taxonomic Resolution Level	Reconstructed Abundance Profile Accuracy
Genus	76.30%
Family	84.22%
Order	87.22%
Class	87.54%
Phylum	87.63%

Female Faecal Abundance Profile - Genus Level

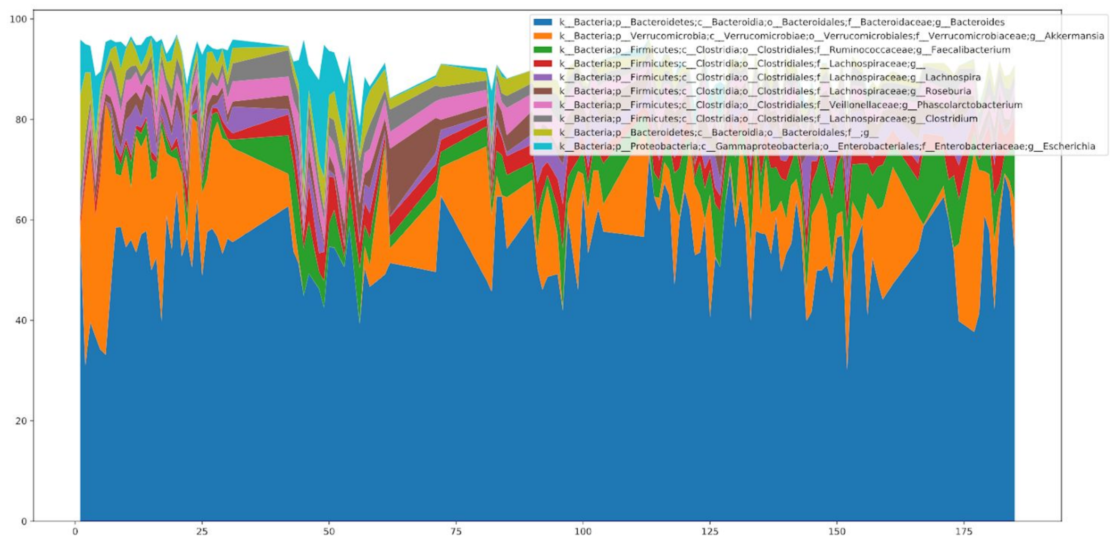

Female Faecal Abundance Profile - Family Level

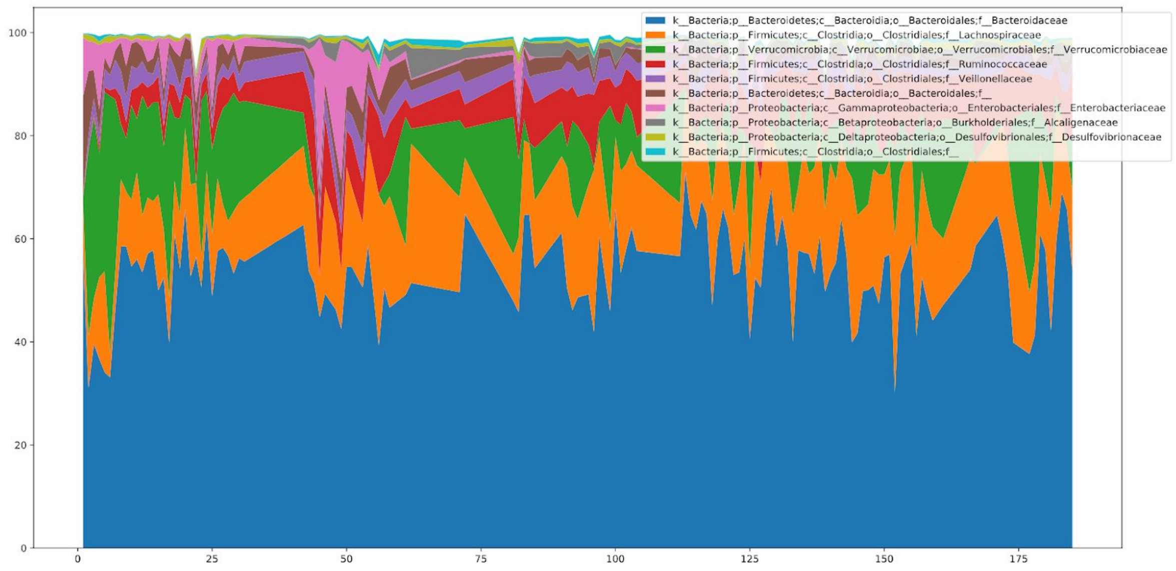

Female Faecal Abundance Profile - Order Level

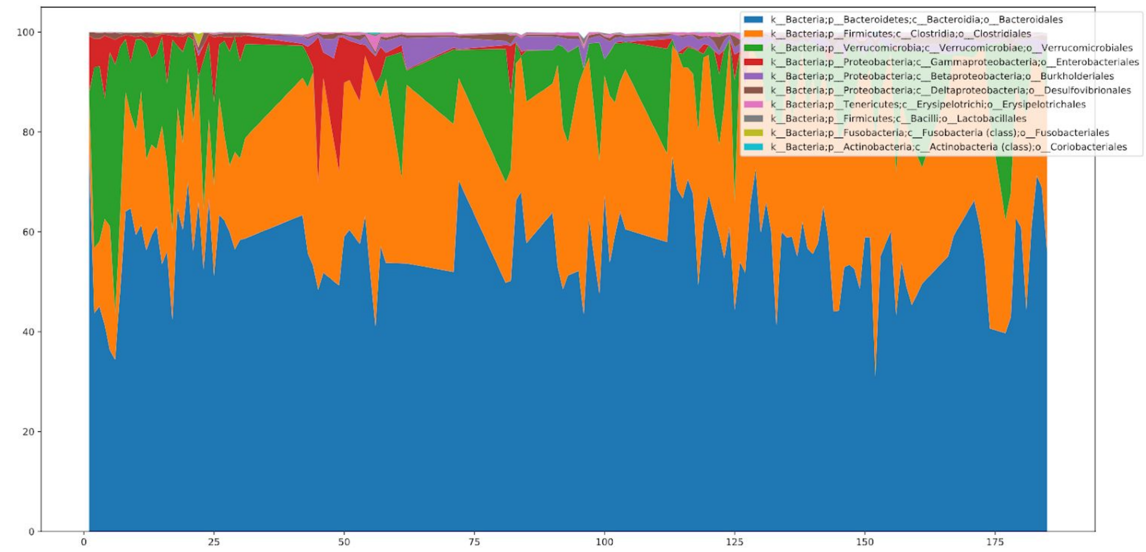

Female Faecal Abundance Profile - Class Level

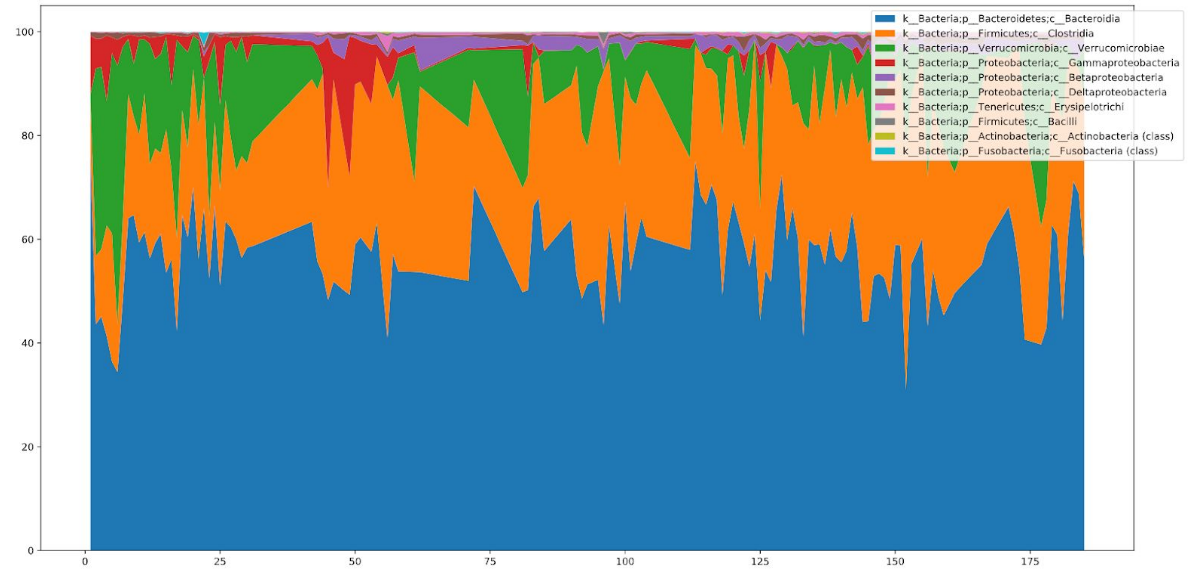

Female Faecal Abundance Profile - Phylum Level

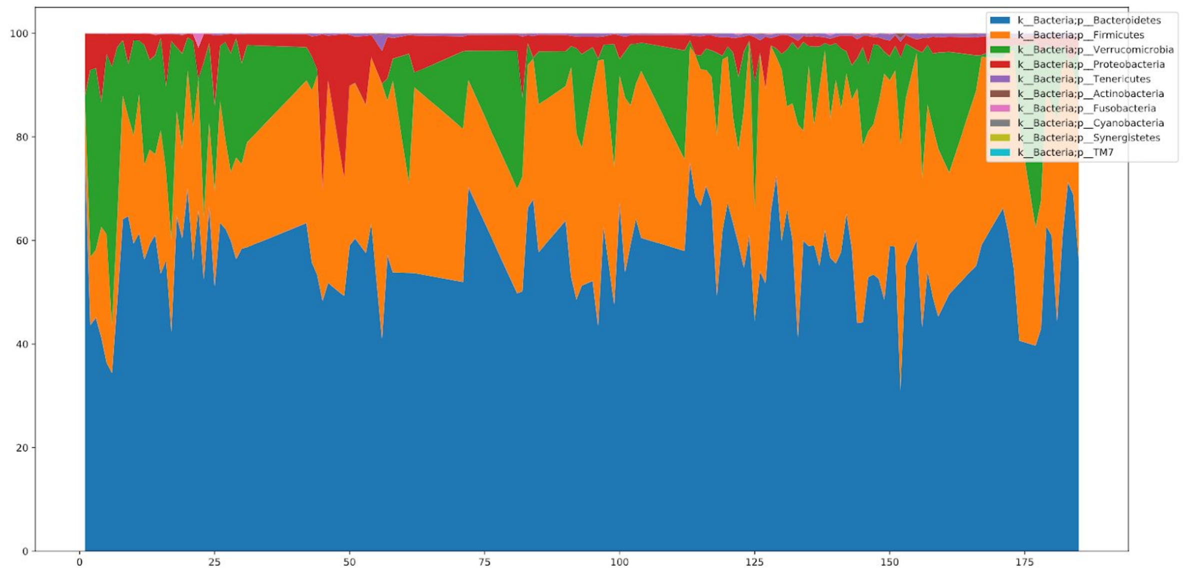

Male Faecal Microbiome Results

No of highest ranking OTUs included	Reconstructed Abundance Profile Accuracy
5	84.16%
10	81.60%
20	78.04%
30	77.96%
40	74.98%
50	77.61%
60	74.95%
70	77.52%

Male Faecal Abundance Profile - Family Level

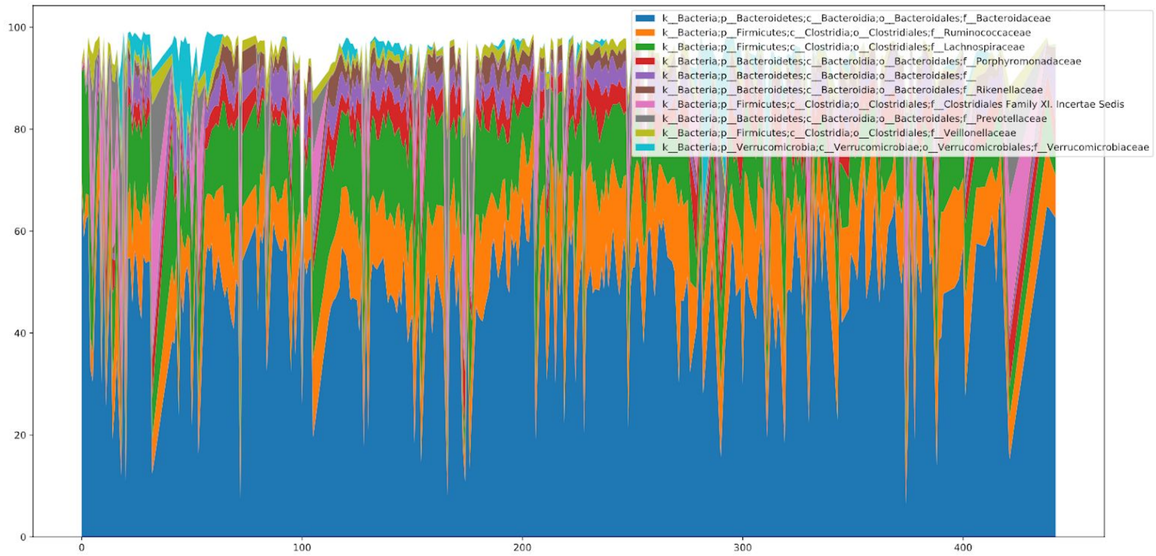

Simulated Data

The number before the abundance profile refer to the instance as shown in the table below.

σ = 1		P				
		p = 0.2	p = 0.4	p = 0.6	p = 0.8	p = 1.0
H	α = 0.2	1	2	3	4	5
	α = 0.4	6	7	8	9	10
	α = 0.6	11	12	13	14	15
	α = 0.8	16	17	18	19	20
	α = 1.0	21	22	23	24	25

1

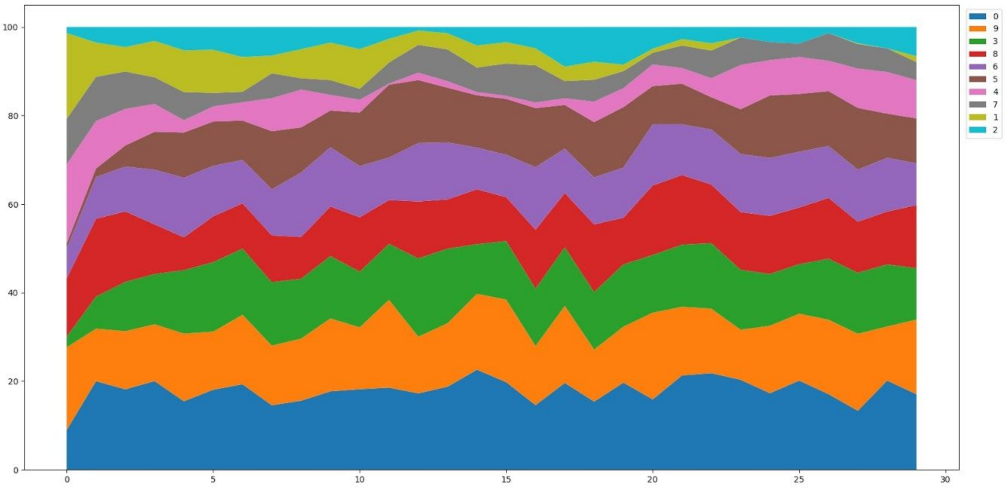

2

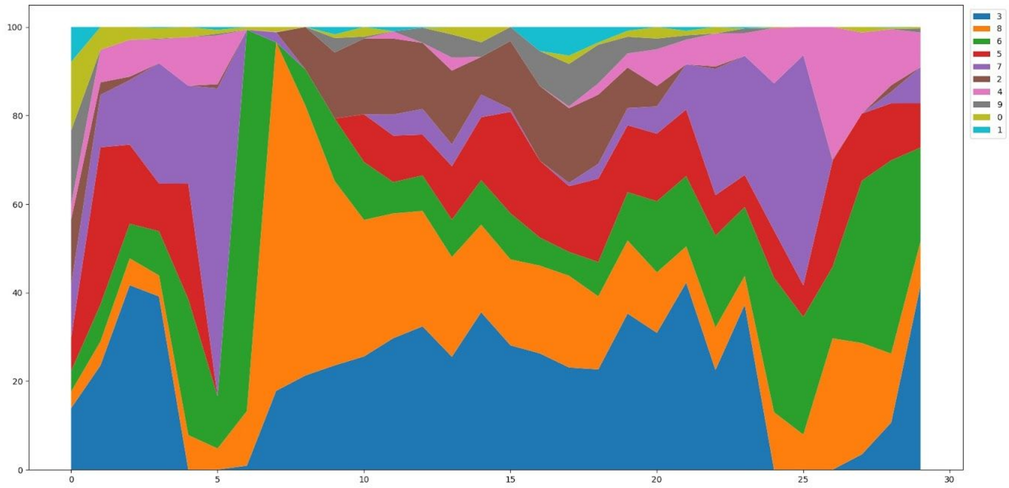

100
80
60
40
20
0
0
5
10
15
20
25
30
3
8
6
5
7
2
4
9
0
1

3

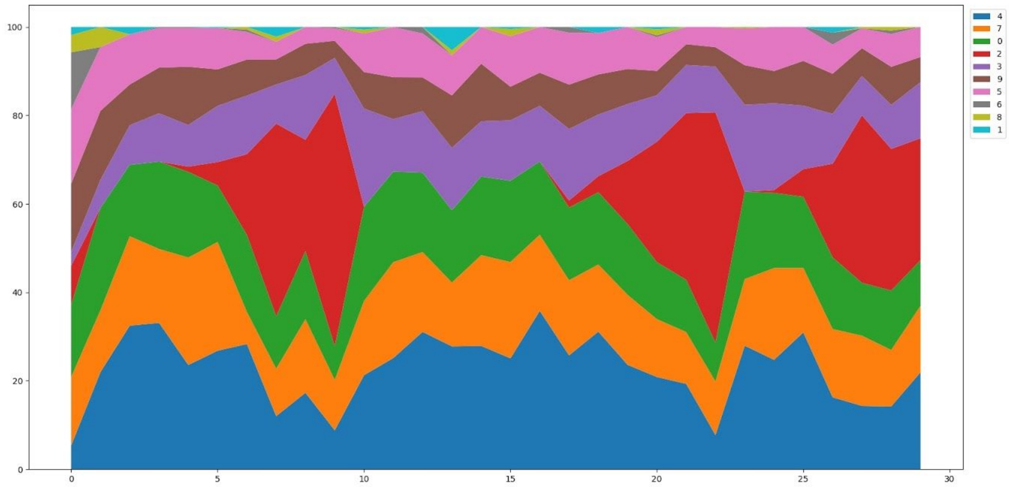

100
80
60
40
20
0
0
5
10
15
20
25
30
4
7
0
2
3
9
5
6
8
1

4

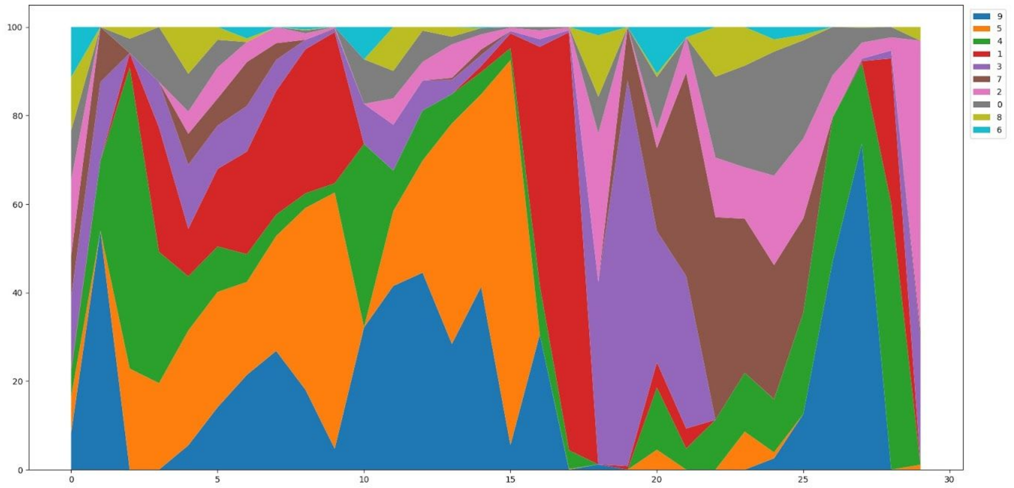
100
80
60
40
20
0
0
5
10
15
20
25
30
9
5
4
1
3
7
2
0
8
6

5

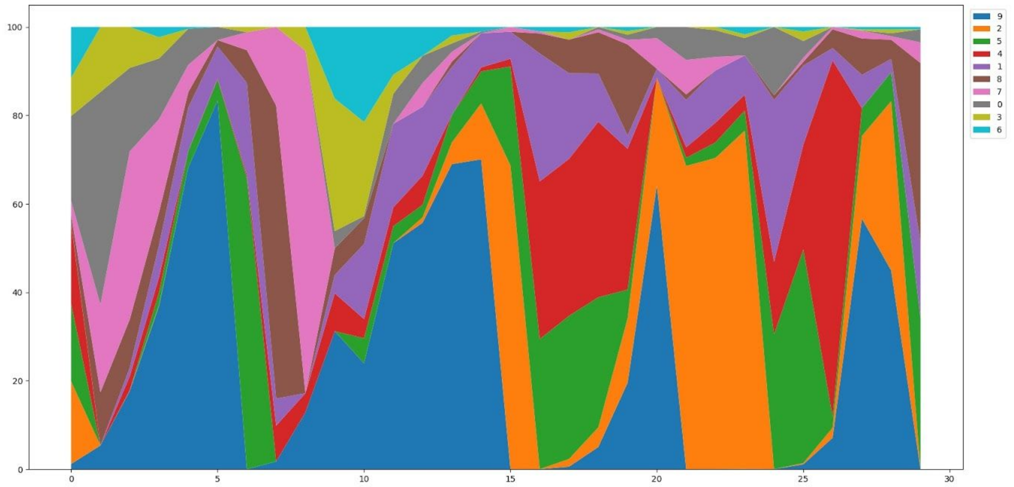
100
80
60
40
20
0
0
5
10
15
20
25
30
9
2
5
4
1
8
7
0
3
6

6

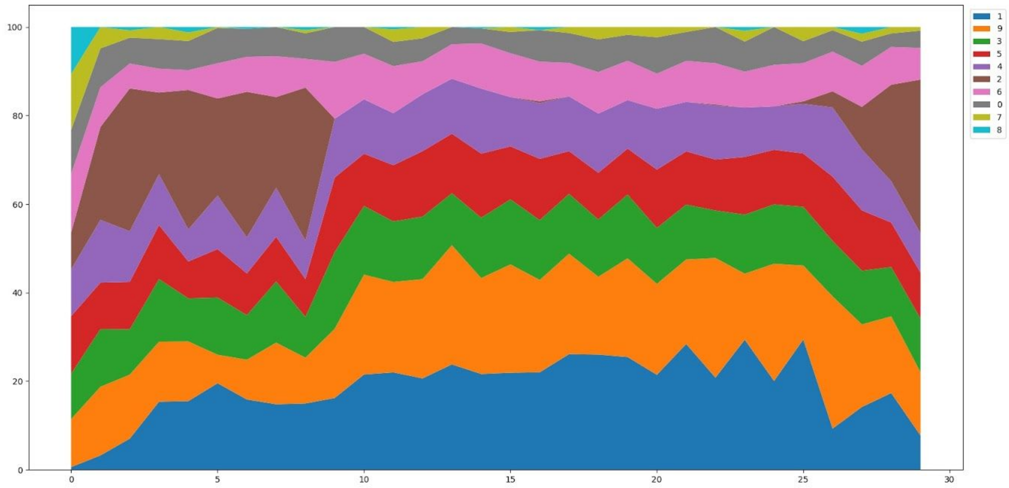

0
20
40
60
80
100
0
5
10
15
20
25
30
1
9
3
5
4
2
6
0
7
8

7

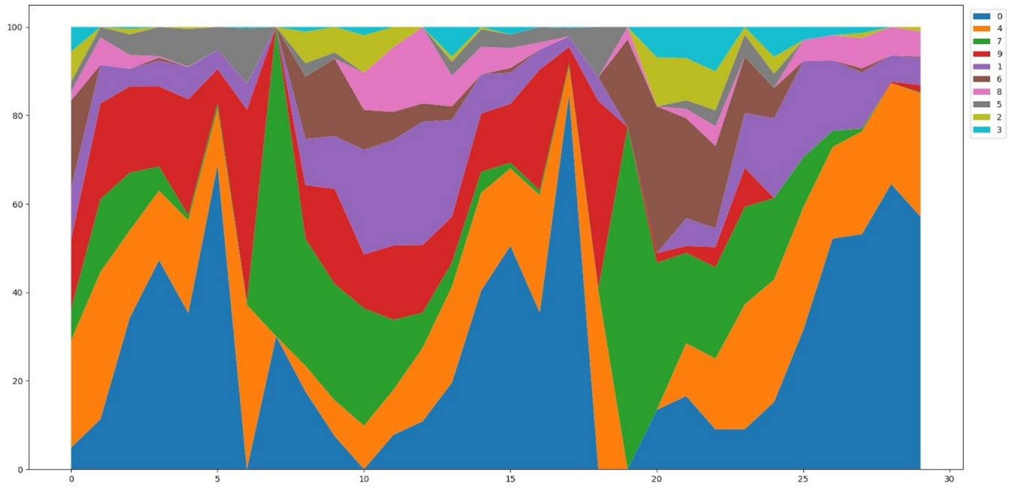

0
20
40
60
80
100
0
5
10
15
20
25
30
0
4
7
9
1
6
8
5
2
3

8

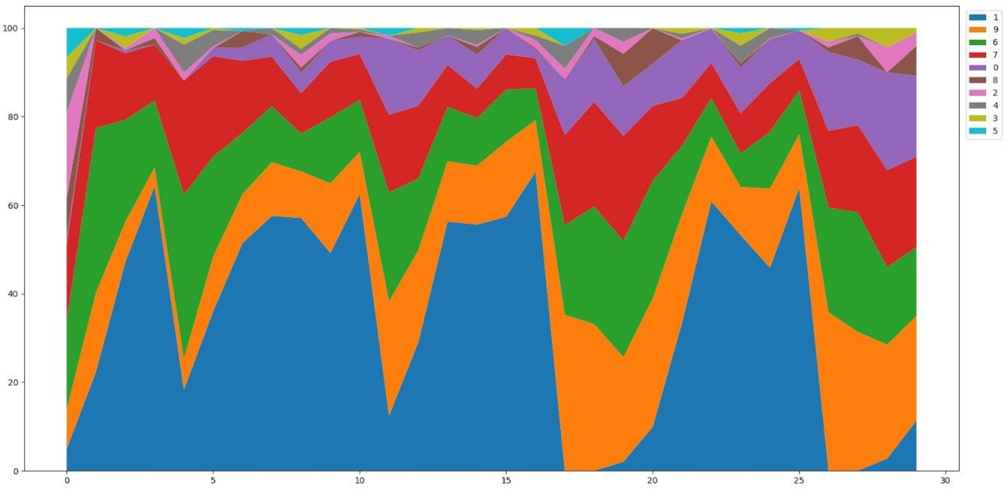

9

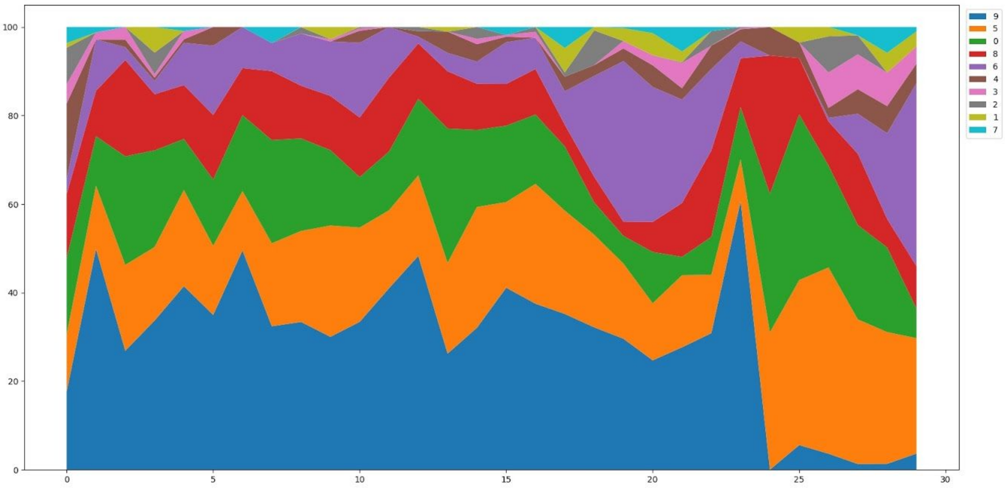

10

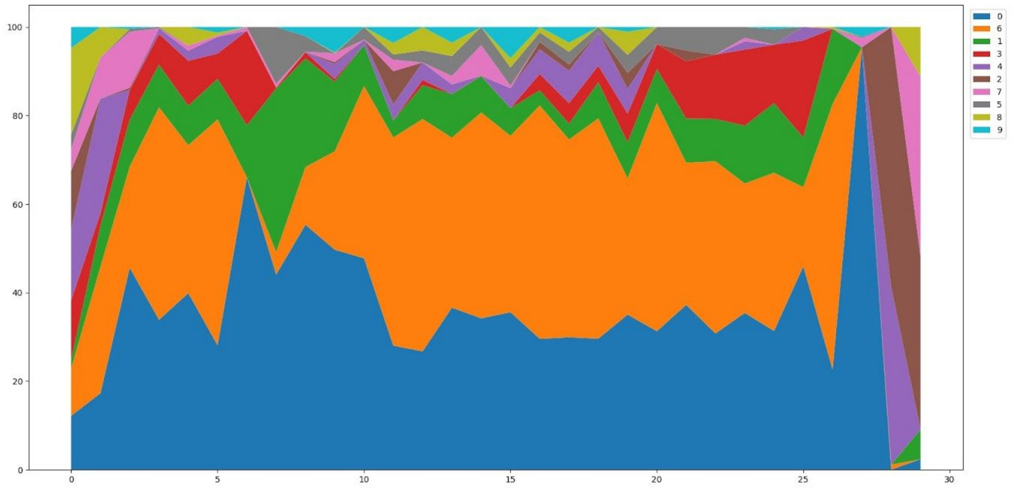
0
6
1
3
4
2
7
5
8
9
100
80
60
40
20
0
0
5
10
15
20
25
30

11

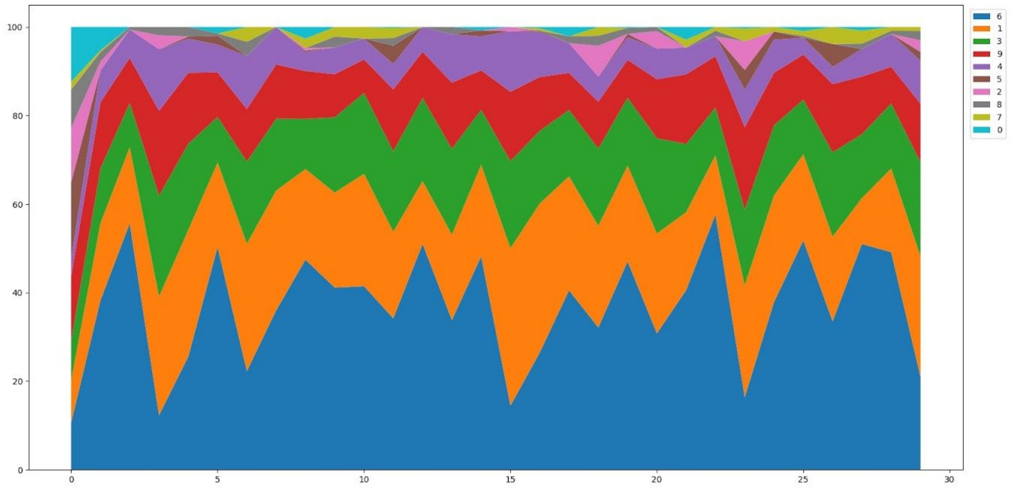
6
1
3
9
4
5
2
8
7
0
100
80
60
40
20
0
0
5
10
15
20
25
30

12

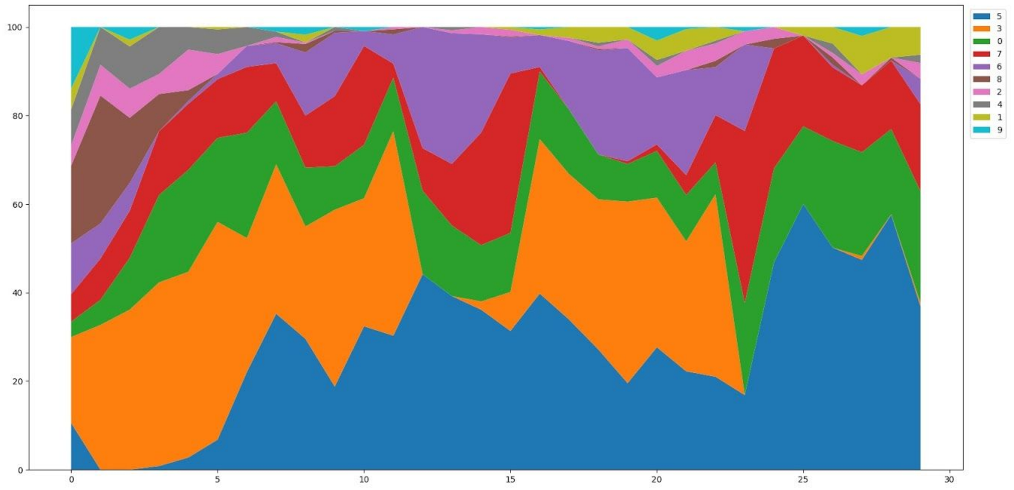
100
80
60
40
20
0
0
5
10
15
20
25
30
5
3
0
7
6
8
2
4
1
9

13

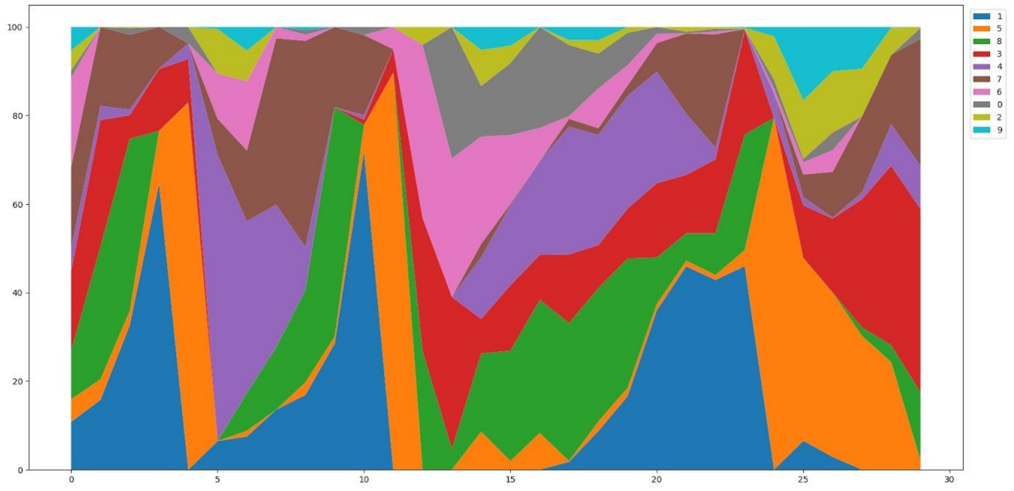
100
80
60
40
20
0
0
5
10
15
20
25
30
1
5
8
3
4
7
6
0
2
9

14

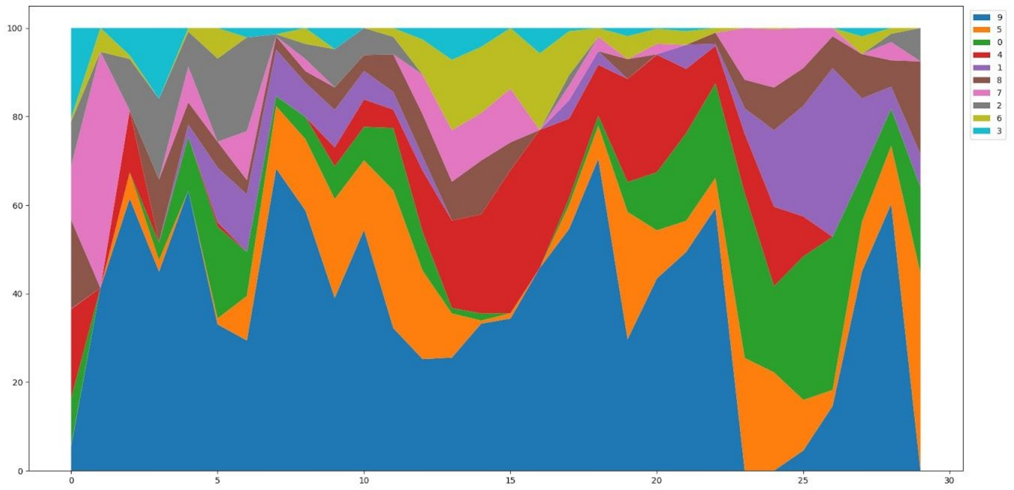

15

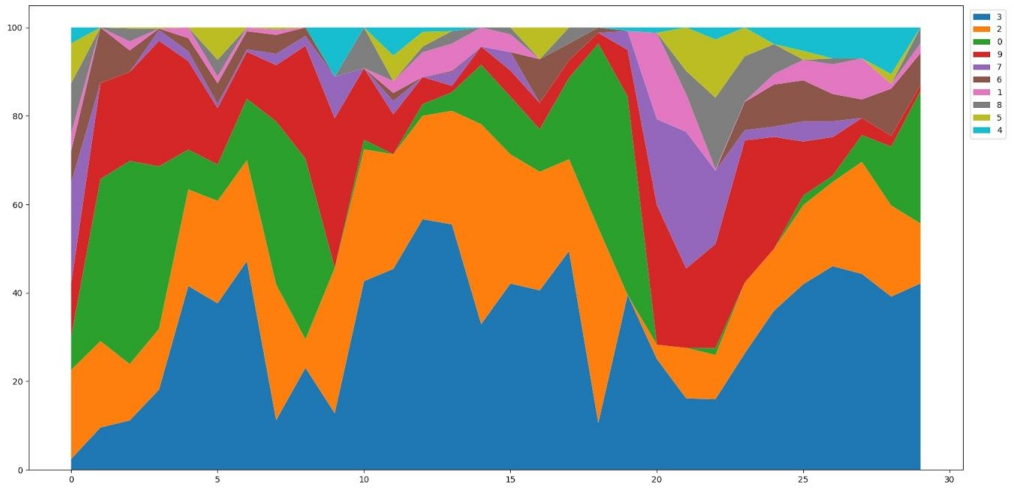

16

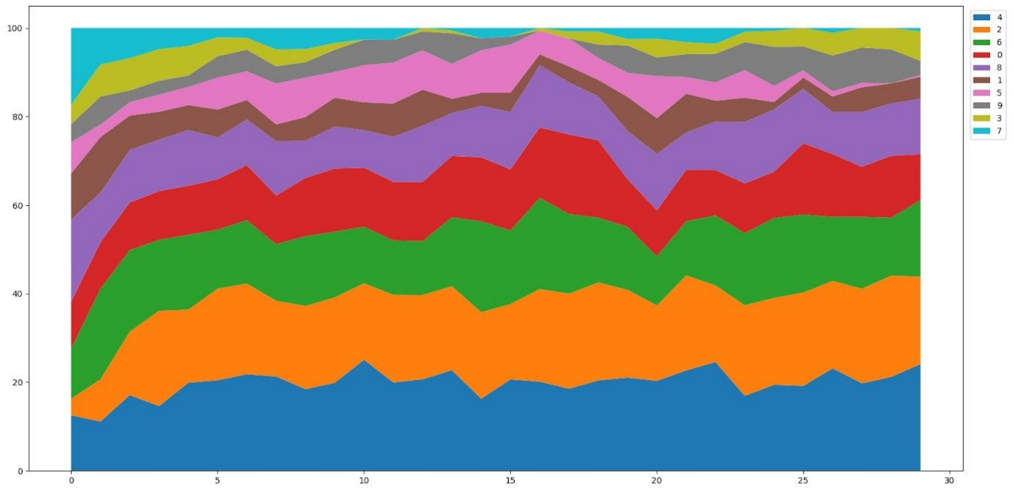

100
80
60
40
20
0
0
5
10
15
20
25
30
4
2
6
0
8
1
5
9
3
7

17

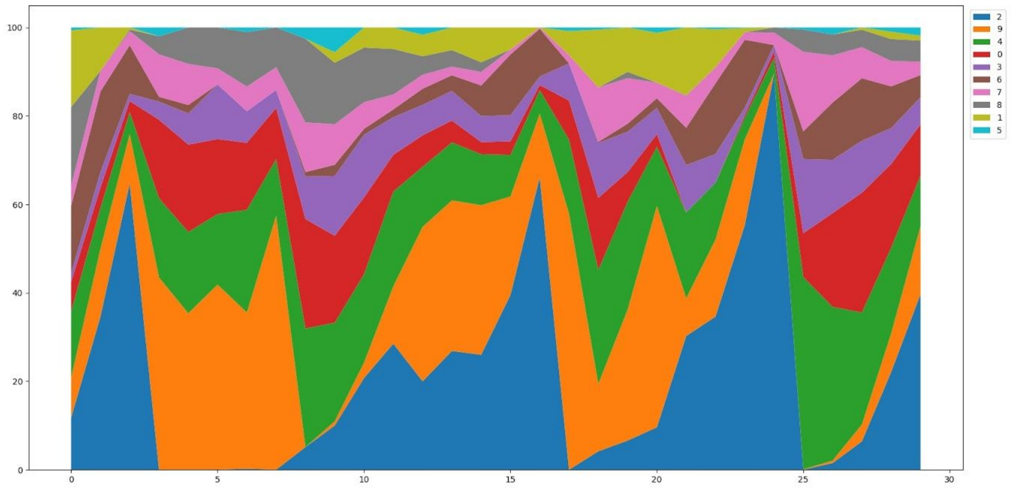

100
80
60
40
20
0
0
5
10
15
20
25
30
2
9
4
0
3
6
7
8
1
5

18

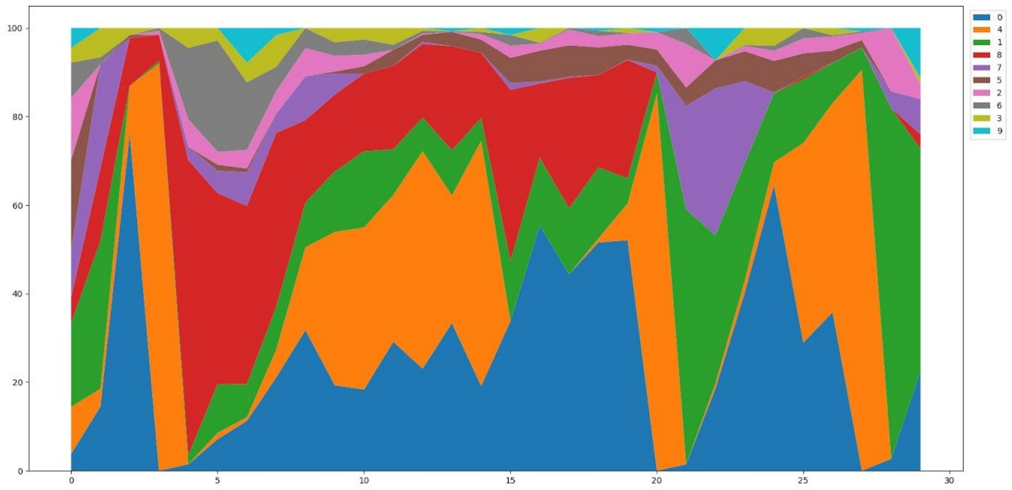

19

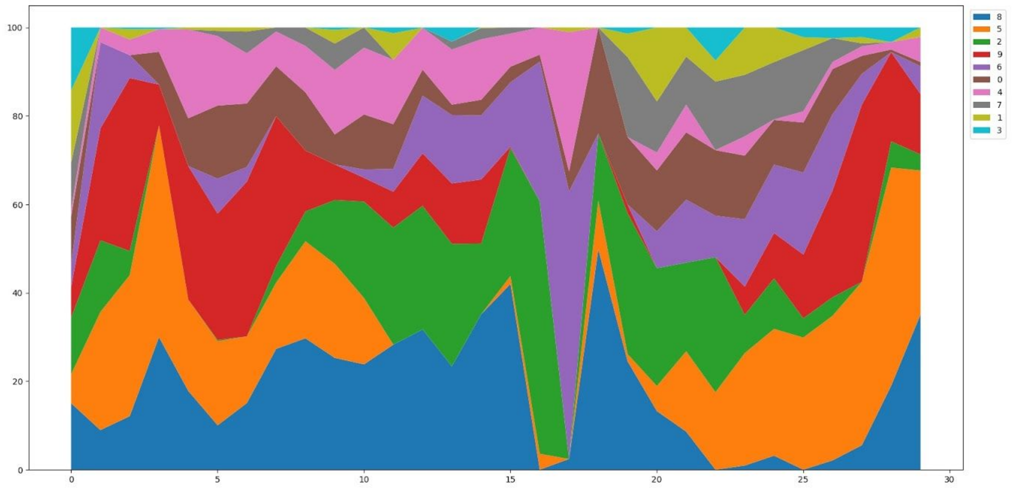

20

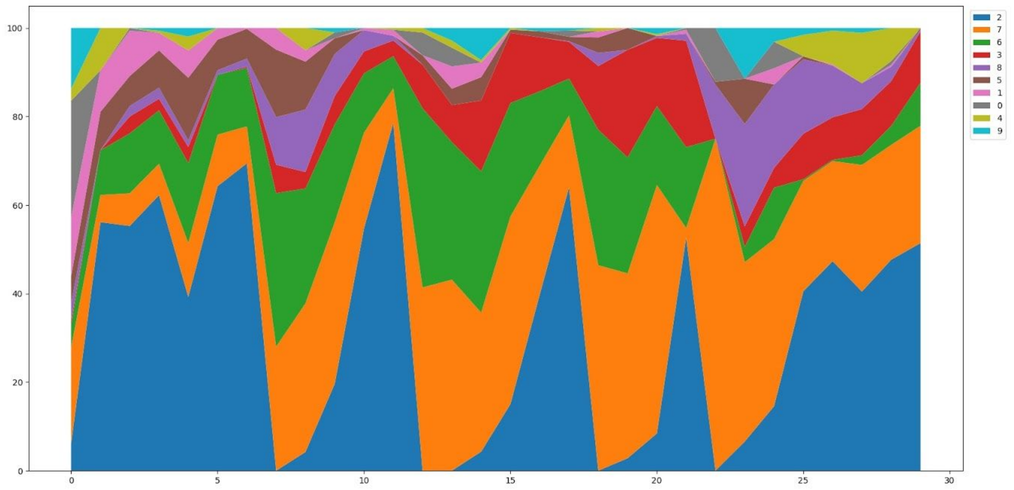

21

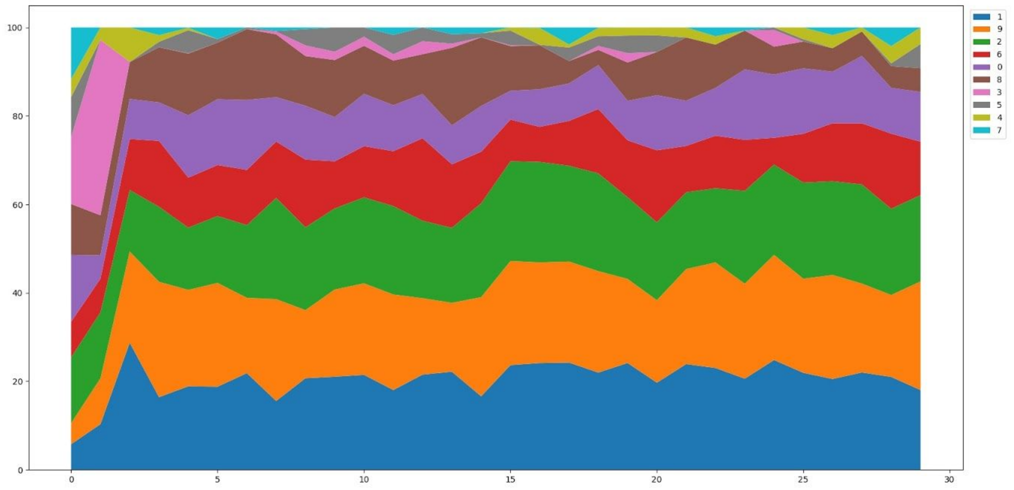

22

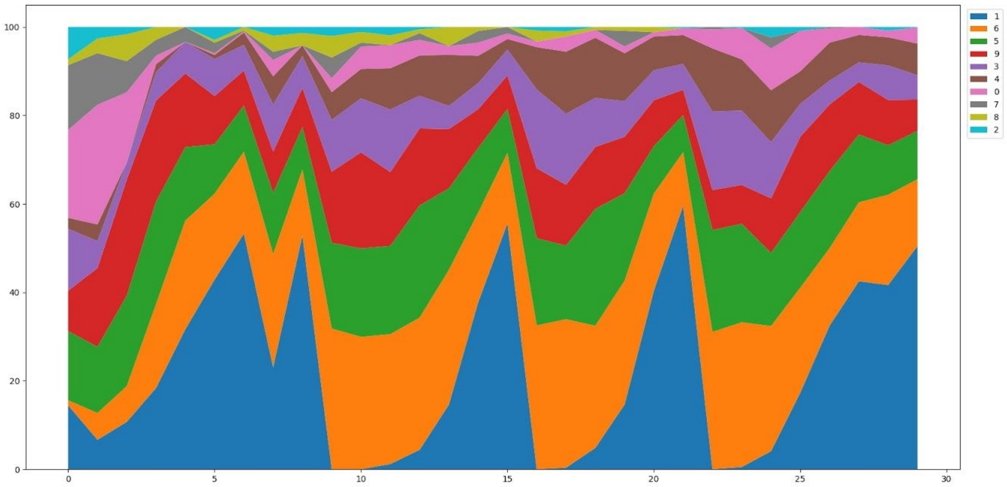

0
20
40
60
80
100
0
5
10
15
20
25
30
1
6
5
9
3
4
0
7
8
2

23

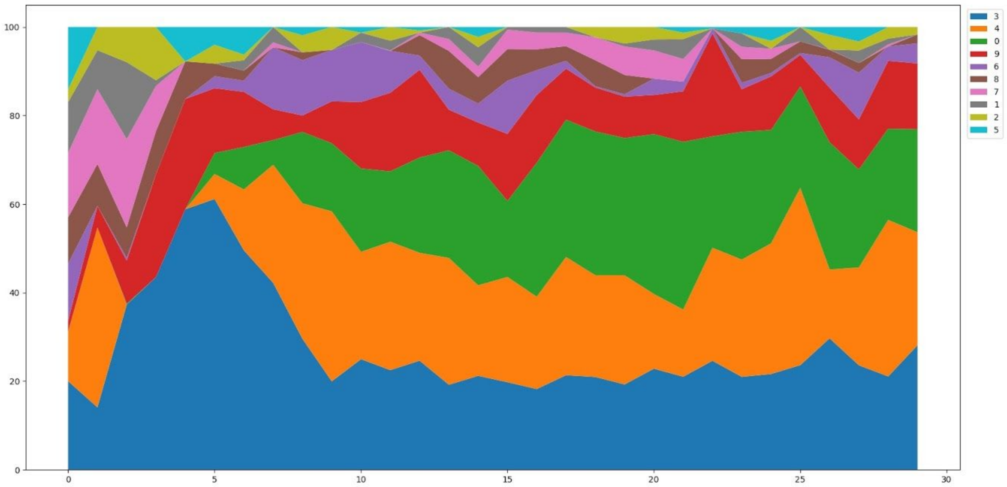

0
20
40
60
80
100
0
5
10
15
20
25
30
3
4
0
9
6
8
7
1
2
5

24

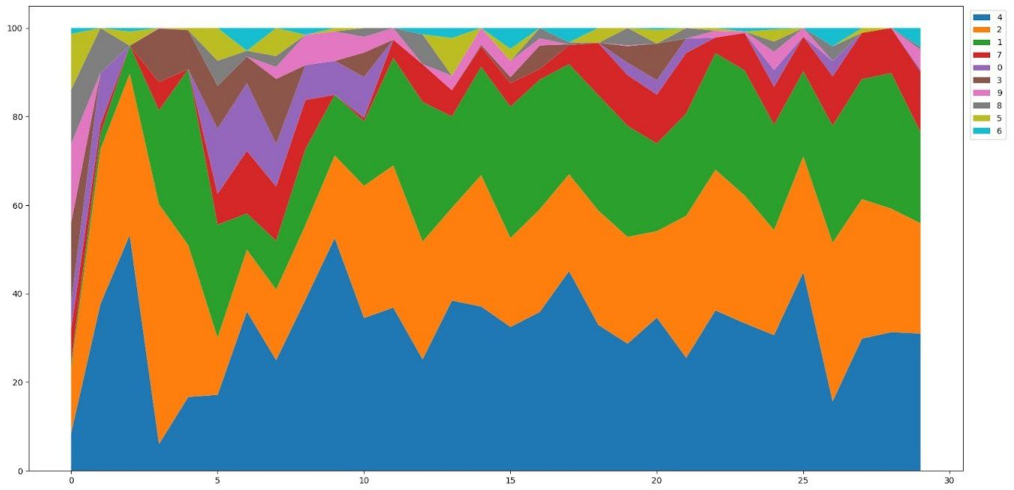
100
80
60
40
20
0
0
5
10
15
20
25
30
4
2
1
7
0
3
9
8
5
6

25

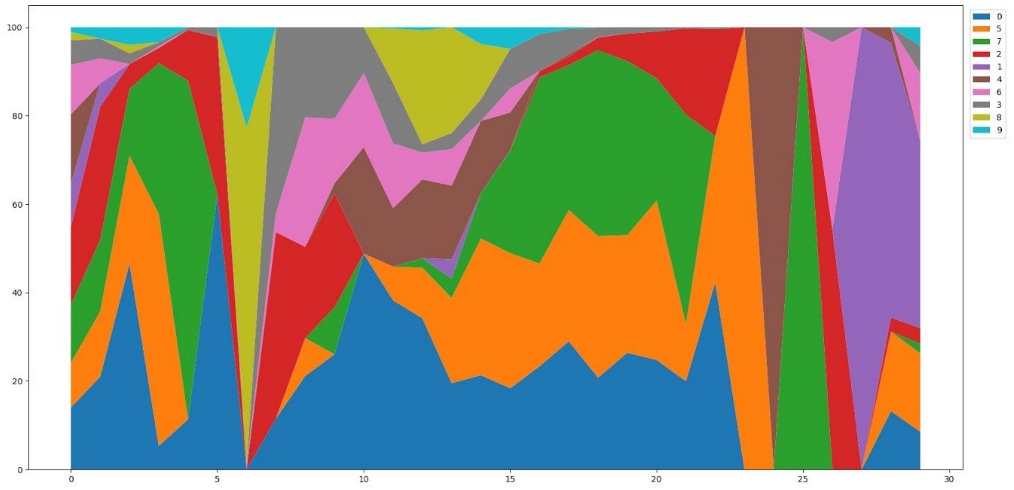
100
80
60
40
20
0
0
5
10
15
20
25
30
0
5
7
2
1
4
6
3
8
9
